# Synergetic effect of bioslurry and charcoal co – composted fertilizers on soil properties and tomato (*Solanum lycopersicum*) productivity

**DOI:** 10.1371/journal.pone.0353951

**Published:** 2026-07-28

**Authors:** Girmaw Mulugeta, Ermias Alayu, Yitayal Addis Alemayehu

**Affiliations:** 1 Department of Environmental Science, College of Agricultural Sciences, Wachemo University, Hosanna, Ethiopia; 2 Kotebe University of Education, Addis Ababa, Ethiopia; University of Agriculture Faisalabad, PAKISTAN

## Abstract

Tomato (*Solanum lycopersicum*) is one of the most widely cultivated and economically important vegetable crops worldwide, contributing significantly to food security. However, its low productivity remains a substantial concern in Ethiopia due to mainly poor soil quality and limited use of inorganic fertilizers. On the contrary, improper disposal of recyclable charcoal and anaerobic cow dung bioslurry (ACDB) wastes poses environmental pollution challenges. Hence, this study aimed to evaluate the synergetic effects of ACDB and charcoal co-composted fertilizers (ACDBCFs) on tomato productivity and soil properties. A pot experiment consisting of eight treatments (T1 - T8) was arranged in a randomized complete block design with three replications. Characterization of the five formulated ACDBCFs revealed good physico-chemical properties of pH, organic carbon, ammonium, nitrate, and orthophosphate, fluctuated from 8.9 to 9.63, 26.8 to 65.6%, 2.5 to 4.6 mgL ⁻ ^1^, 11.9 to 18.2 mgL ⁻ ^1^, and 0.74 to 3.1 mgL ⁻ ^1^, respectively. Significant differences (p < 0.05) were observed in tomato fruit yield with relatively higher values of 2.87 and 2.73 kgpot ⁻ ^1^ for T6 and T8 under ACDB + charcoal (+10%) and inorganic fertilizers amendments, respectively. Principal component analysis (PCA) demonstrated strong associations between nutrient availability and tomato growth and yield component parameters. Heat map visualization further confirmed that ACDBCFs application improved soil background nutrient and carbon contents. Overall, the findings from this short-term study indicate that ACDBCFs can enhance tomato productivity while improving soil organic matter and nutrient availability. However, further long-term studies on field level validation in real soil conditions and nutrient dynamics are recommended to support large-scale adoption of this resource-recovery approach, contributing to sustainable agriculture, environmental protection, and socio-economic development.

## 1. Introduction

Tomato (*Solanum lycopersicum*) is an important nutritional crop in many parts of the country, including Ethiopia, contributing to smallholder livelihoods. However, its average national potential yield (12.5 t ha^-1^) remains low as compared with the neighboring Sub-Saharan African countries such as Kenya (16.4 t ha^-1^) [1]. This yield is mainly limited by factors like poor soil fertility and limited use of inorganic fertilizers [2]. Loss of agricultural soil fertility is one of the major constraints limiting vegetable production in Ethiopia, particularly in smallholder systems where access to inorganic fertilizers is restricted by high cost and inconsistent supply [3]. This implies that sustainable agricultural practices are increasingly needed in Ethiopia to ensure resilient agriculture. Organic amendments such as compost, anaerobic cow-dung bioslurry (ACDB), and other locally available wastes have gained attention for resilient agricultural practices through improving soil health [4], and through supporting essential plant nutrition, and mitigating plant stresses [5]. ACDB contains easily decomposable organic matter and essential nutrients that enhance soil structure, microbial activity, nutrient cycling, and plant growth [6,7]. It is also reported that the use of ACDB as fertilizer produces higher tomato yields than farmyard manure [8]. In the same argument, utilization of ACDB in Ethiopia also shows enhanced vegetable production through improving soil fertility [9,10]. Despite its importance, the direct use of ACDB alone often has a low organic carbon content, carbon to nitrogen ratio, and high pH, which hinder its potential uses [11,12]. Furthermore, ACDB easily loses its nitrogen content through ammonia volatilization and requires co-digestion with other eco-friendly substrates that have the potential to retain nutrients [13,14]. This highlights a need for selecting effective adsorbents that recover nutrients from ACDB while reducing environmental contamination.

Biochar or charcoal, a carbon-rich and porous material, produced by pyrolysis of organic substances under anaerobic conditions [15], which serve as a good candidate for nutrient retention and can improve nutrient availability, increase water-holding capacity, soil aeration, carbon sequestration, and reduce greenhouse gas emissions and nutrient leaching [16–18]. Its partial combination with ACDB is tested in Ethiopia, and shows promising outcomes in improving soil structure, water retention, and nutrient-use efficiency, especially in degraded tropical soils [2]. In addition, it enhances nutrient stability, increases exchangeable cation capacity, and supports higher crop yields [12], and creates a synergistic effect on soil physico-chemical and biological characteristics [19] and plant growth quality [20]. Moreover, a recent study reported that biochar- based organic fertilizers (BBOFs) enhance the degraded soil’s physico-chemical characteristics [21]. In general, pilot scale study evidences revealed that BBOF applications in degraded soils improve soil fertility [22]. Studies outside Ethiopia also recommended biochar as a promising nutrient adsorbent that reduces nutrient leaching and releases nutrients slowly to plants [23,24]. Despite the availability of fine powdered charcoal residual wastes in many urban areas, there is limited empirical evidence on their application as agricultural inputs in combination with ACDB on tomato productivity and soil properties. Furthermore, low soil fertility is becoming a big challenge in Ethiopia, where continuous cultivation, limited organic inputs, and heavy reliance on inorganic fertilizers have degraded soil productivity and reduce vegetable production [2]. In our study area, Hadiya zone, central Ethiopia, declining soil organic matter and nutrients affect the sustainable production of cereals and vegetables. Hence, testing different combination ratio of ACDB and charcoal co – composted organic fertilizers (ACDBCFs) is needed to understand their synergetic effects on vegetable productivity and soil fertility. Therefore, this study aimed to assess how ACDBCFs influence *Solanum lycopersicum* productivity and soil properties. Specifically, the study sought to: (1) analyze the physico-chemical characteristics of both ACDB and charcoal wastes; (2) evaluate the effects of ACDBCFs applications on tomato biometric data; and (3) assess the short-term residual impacts of ACDBCFs on slightly acidic soil properties.

## 2. Materials and methods

### 2.1. Experimental site description

The pot experiment was conducted in a greenhouse system at Ambicho Kebele, Lemo Woreda, Hadiya zone, central Ethiopia region, which is located southwest of Addis Ababa at a distance of 230 km. Geographically, the study area, Ambicho Kebele is located between 7°38’26.8"N and 37°58’18.7"E with an altitude of 2300 m.a.s.l. The area receives a mean annual rainfall of 900–1400 mm and a temperature between 13 °C and 23 °C. In the region, mixed agriculture (crop and livestock) is the main livelihood of the community. Tomato (*Solanum lycopersicum*) is one the major cultivated and widely consumed vegetable in the study area [25]. The necessary experimental raw materials; charcoal wastes were collected from twelve locations of Hosanna town: Naremo Kela, Naremo condominium, Sinodos Kela, St. Mariyam, Stadium, Saturday Market, 18 taxi mazoria, 18 Tekelhaimanot, Arada, Soro Bere, hospital, and Wanza village. The samples were homoginized and coarse charcoal samples were chopped to enhance surface area and the mass was measured using a beam balance and stored in a polyethylene bags for further uses. Similarly, the ACDB was also collected from Ambicho Kebele, Lemo Woreda farmers’ anaerobic digester village using a four polyethylene bottles with holding capacity of 5 Liter from 8:00 a.m. to 9:00 a.m. and stored in refrigerators overnight. Then after, both were subjected for formulation of five different types of ACDBCFs.

### 2.2 Formulation of ACDBCFs

Five different ACDBCFs were prepared from predetermined mass ratios using a co-composting approach. Charcoal quantities were calculated by adjusting the recommended application rate of 5.5 t ha ⁻ ¹ by −5%, −10%, 0%, + 5%, and +10% [26]. A constant mass of 0.31 kg of ACDB, determined from the recommended application rate of 5 t ha ⁻ ¹ [27], was used for each treatment. The corresponding charcoal masses were then mixed with the ACDB according to the designated ratios. The mixtures were co-composted for 30 days in polyethylen plastic composting containers in the Environmental science laboratory of Wachemo University. During co-composting, the materials were periodically mixed using a stainless steel to ensure aeration and uniform homogenization. After the co – composting period, the prepared ACDBCFs were air-dried for one week, ground into fine powder, weighed, and stored in polyethylene bags for subsequent use for pot experiments. The detail formulation ratios of the treatments are presented in Table 1.

**Table 1:** ACDBCFs formulation stoichiometric ratio.

Treatment types	Description	Amount of charcoal (kg)	Amount of ACDB (kg)
ACDBCF1	ACDB + charcoal (−5%)	0.33	0.31
ACDBCF2	ACDB + charcoal (−10%)	0.31	0.31
ACDBCF3	ACDB + charcoal (recommended)	0.35	0.31
ACDBCF4	ACDB + charcoal (+5%)	0.37	0.31
ACDBCF5	ACDB + charcoal (+10%)	0.38	0.31

### 2.3 Experimental design, treatments, and operations

**Fig 1:**
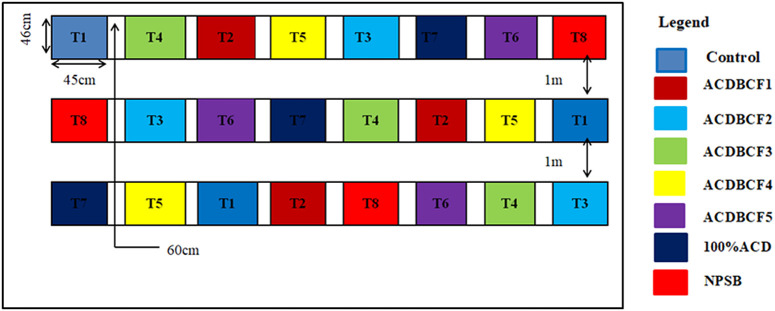
Design of the pot experimental system and treatments.

A pot experiment was carried out in a randomized complete block design (RCBD) with eight treatments in three replications at a pot area size of 45 cm × 46 cm in a greenhouse system, designed in accordance with Berghage [28], which size was 10 m by12 m. The treatment details are indicated in Fig 1 and Table 2.

**Table 2:** Total amount of ACDBCFs, ACDB, and NPSB applied per pot.

ACDBCFs types	Amount applied (kgpot^-1^)
T1 (Control without any treatment)	0
T2 (ACDB + charcoal (−5%) treated)	0.208
T3 (ACDB + charcoal (−10%) treated)	0.214
T4 (ACDB + charcoal (recommended) treated)	0.220
T5 (ACDB + charcoal (+5%) treated)	0.226
T6 (ACDB + charcoal (+10%) treated)	0.232
T7 (100%ACDB treated)	0.104
T8 (NPSB treated)	0.002

Before seedling, each pot was filled with 3 kg soil collected from uncontaminated areas, and the different ACDBCFs were incorporated at application rates of 5 t ha^-1^ as indicated in Table 2, and allowed to stabilize for two weeks. Then after, each pot was moistened initially with 2 liters of potable water. Local *Solanum lycopersicum* variety, which has better adaptation, high-yielding performance, and disease resistance was used as a test crop [1]. A four healthy seeds of tomato were sown in each pot, and 100 kg ha^-1^ NPSB was applied under T8 pots [29], and each treatment pot was watered with 2 liters of potable water for the first 2 weeks, and two tomato seedling were left per pot for the main experimental test. The watering was also continued at the maturity and flowering period every day with one liter. Whereas, during the fruiting period, one liter was watered in a two-day interval using a spray bucket from 8:00 a.m. to 9:00 a.m. Normal tomato crop agronomic practices such as trimming leaves and tying up tomato plants with wood stakes, and weed control were performed.

### 2.4 Charcoal, ACDB, and ACDBCFs samples collection and analyses

#### 2.4.1 Charcoal waste collection, preparation, and analysis.

The unwanted materials were removed by hand and sieving from the collected charcoal samples and charcoal samples were mixed in equal proportions. The composite coarse charcoal sample waste was further processed to increase its purity and homogeneity through washing with distilled water and kept for sun drying. The sun-dried charcoal sample was oven-dried at 105 ^°^C for 24 hours and cooled. The cooled charcoal sample was ground into a fine powder of < 2 mm size and stored in a polyethylene bags for experimental testing. From the stored samples, a 1 kg charcoal sample was taken and subjected for the measurement of pH, electrical conductivity (EC), ash content, moisture content, pore volume, bulk density, volatile organic matter, carbon content (OC), and carbon: nitrogen ratio using standard procedures. The charcoal sample pH and EC were measured from a 1 g (fine powdered charcoal sample): 2.5 (distilled water) suspension [30]. The moisture content, ash content, volatile organic matter, and OC contents were determined using standard procedures [31]. The bulk density and pore volume were determined following Verla et al. [32] procedure.

#### 2.4.2 Characterization of ACBD and ACDBCFs.

ACDB was collected from an anaerobic cow dung biogas digester of a farmer’s residence from Lemo Woreda at one time using a 1 Liter sterile glass bottle from the sampled 5 liters volume and transported to the laboratory using refrigerated bags for immediate analysis, and production of ACDBCFs. The ACDB and ACDBCFs pH and EC were measured using pH and EC meters, respectively [33]. The OC of ACDB and ACDBCFs were determined by the Wakely and Black method [34]. Total phosphorus (TP) and orthophosphate (PO_4_– P) were determined using sodium bicarbonate (NaHCO_3_) and ascorbic acid methods, respectiveely using spectrophotometer (UV-2600, Shimadzu, Japan) [35]. The total nitrogen (TN) content was determined using the persulfate digestion method [36]. Whereas, the NH_4_ – N and NO_3_ – N ions were determined using the APHA method [33]. Carbon to nitrogen ratio was calculated following Kiflu et al. [37]. ACDB and ACDBCFs exchangeable cations such as sodium (Na^+^), potassium (K^+^), calcium (Ca^2+^), and magnesium (Mg^2+^) ions were determined with acid digestion (1 g samples was placed in 25 ml beakers and next, 10 mL 69–70% HNO_3_ and 10 mL 35.4% HCl were added along with 30% H_2_O_2_) using Atomic Absorption Spectrometer (Shimadzu AA-7000, Japan). Concentrations of trace elements such as zinc (Zn^2+^), copper (Cu^2+^), iron (Fe^2+^), and manganese (Mn^2+^) ions were also determined with acid digestion method [33].

### 2.5 Measurement of tomato biometric data

The tomato growth component parameters such as number of leaves per plant, plant height, and stem diameters were measured at seedling stage (20 days after germination), at mild growing stage (55 days of germination), and at maturity stage (95 days of germination) following standard procedures. Furthermore, the tomato fresh weight and dry weight biomass yields were determined in kgpot^-1^ using standard procedures [38].

### 2.6 Soil sample collection and analysis before and after the experiments

Representative soil samples were collected from each treatment pot before and after tomato plant harvesting using a stainless steel scoop from the top layer to the bottom layer of the pot, and composited in a bucket. A 1 kg composite soil samples were air dried, ground into less than 2 mm sieve size, and stored in polyethylene bags for laboratory analysis. The soil moisture content was determined using oven-dry methods [39]. The pH and EC were measured using Mebrahtom [33]. OC content of soil was determined by the Wakely and Black method [34]. TP and PO_4_– P were determined using sodium bicarbonate (NaHCO_3_), and ascorbic acid methods, respectively [35]. The TN content was determined using the persulfate digestion method [36], while the NH_4_ – N and NO_3_ – N and exchangeable cations (Na^+^, K^+^, Ca^2+^, and Mg^2+^) were determined using the APHA method [33]. Carbon to Nitrogen ratio was calculated from the determined carbon and nitrogen values [34].

### 2.7 Experimental quality control and data analysis

This study doesn’t involved human, animal, and environmental issues, however, ACDB and charcoal samples collection was done through obtaining free and informed consent from Lemo Woreda farmers and Hosanna town charcoal shopping centers, respectively. After that, all samples and experimental procedures were carried out using pre-cleaned, sanitized high-density plastic bottles and suitably labeled containers together with analytical-grade chemical reagents to guarantee analytical precision and repeatability. All subsequent tests were performed following the allotted regulatory deadline, and sample collection and preservation closely followed established standard practices. Triplicate analysis of every sample and regular equipment calibration were used to maintain quality assurance and control measures. Analysis of Variance (ANOVA – one way including Turkey and Dunn Sidak tests) was used to assess treatment effects and data variances at 5% significance level using OriginPro 2026 [40]. Principal component analysis (PCA) was used in accordance with standard techniques to investigate multidimensional data structures and reduce variable dimensionality [41]. Additionally, heat map was performed to visually profile patterns and correlations within the soil-plant matrix using established multivariate visualization guidelines [42]. The data obtained from the pot experiment and laboratory-based analysis were analyzed and summarized into tables and graphs.

## 3. Results and discussion

### 3.1 Physico-chemical properties of charcoal

**Fig 2:**
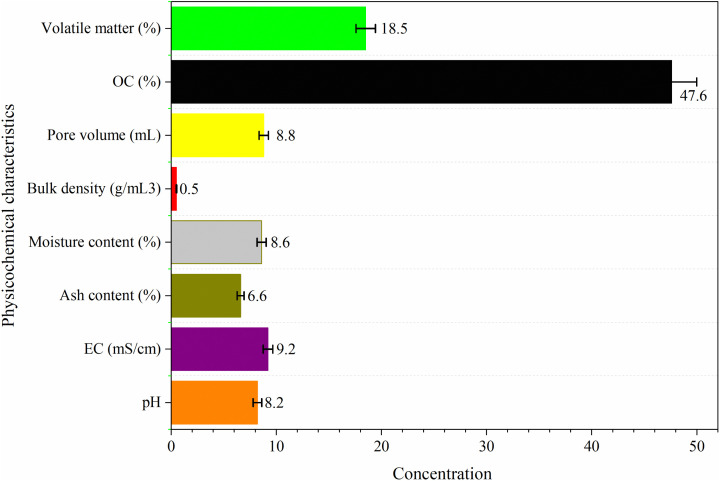
Physico-chemical characteristics of charcoal waste.

The charcoal’s physico-chemical characteristics are indicated in Fig 2. These physico-chemical data suggests that the charcoal is suitable for soil amendment and agricultural applications because of its comparatively low ash, bulk density, volatile matter, and moisture contents, as well as its high organic carbon content, and mild alkaline properties. The charcoal’s alkaline pH of 8.2 ± 0.41 suggests that it could help raise the pH of acidic soils. According to earlier research evidence, the pH of biochar typically ranges from 3.1–12.0 [43], depending on the type of feedstock and the pyrolysis temperature. However, for acidic soil amendments, the biochar pH value should be from 8.84–9.84 [44]. The charcoal pH value found in this study is close to this standard value, and can be used in producing a good ACDBCFs, which may serve as a potential alternative for acidic soil amendment, as it could maintain the acceptable pH range (6.0–7.5) for most plants [45]. This higher pH value may be associated with the higher temperatures used for charcoal production, which often result in more alkaline materials because of greater ash production and alkaline mineral concentration [46]. Thus, the observed pH value indicates a possible liming impact on acidic soils and is within the permissible range stated for high-quality biochars. The EC value, 9.2 ± 0.46 mScm ⁻ ¹, also suggesting a moderate level of soluble salts present in the charcoal, and is found in the recorded EC value range of 0.04–54.2 mScm^-1^ [43]. Elevated EC values in biochar are typically linked to the concentration of inorganic nutrients like potassium, calcium, and magnesium during carbonization. Research evidence has shown that EC generally increases with increasing pyrolysis temperature and ash content because mineral constituents become concentrated when volatile compounds are removed [47]. The relatively high EC found in this study suggests that the charcoal can be attributed to ACDBCFs content of soluble exchangeable cations and anions, which are relevant for certain agricultural applications as a source of plant nutrients [44]. Furthermore, the charcoal moisture and ash contents were 8.6 ± 0.43% and 6.6 ± 0.33%, respectively, which are relatively low compared to the earlier reported biochar moisture and ash content values of 5.3 and 6.2%, respectively [48], depending on feedstock and manufacturing conditions. Ash, which is the inorganic mineral component left over after combustion, has a major impact on the alkalinity and nutritional availability of biochar. This study’s modest ash percentage shows that the charcoal preserved enough mineral components while retaining a significant carbon fraction, which is ideal for soil conditioning and carbon sequestration. In terms of the moisture content, the low moisture value is beneficial because it increases nutrient retention and transportation efficiency, decreases microbial deterioration during storage, and improves storage stability [46]. In general, the measured pH, EC, moisture and ash content qualities show that the charcoal has good suitability for application in agriculture.

The charcoal’s bulk density (0.5 ± 0.02 gcm ⁻ ^3^) indicated in Fig 2, also showed promising characteristics. This higher pore volume might be associated with the increase in charcoal pore size distribution and surface structural changes, which lead to enhanced water holding capacity and nutrient retention [49]. Furthermore, it plays essential role in soil nutrient transportation, integration, and porosity. The present study’s charcoal bulk density is lower than the reported bulk density (0.74 g/mL^3^) of leaves of *Cordia africana* [50]*,* but the bulk density value falls in the range of 0.37 to 0.65 g/mL^3^ [44]. The bulk density variations might be due to variation in feedstock and pyrolysis conditions [51].This low-density charcoal increase soil water-holding capacity, decrease soil compaction, and enhance soil aeration. Greater carbonization and structural compaction during pyrolysis may be linked to the somewhat increased bulk density found in this investigation [52]. The charcoal has also a well-developed porous structure (8.8 ± 0.44 mL), which might be due to the elimination of volatile matters and the formation of different pore sizes. One of the key factors influencing the agronomic and environmental performance of biochar is its porosity. High pore volume improves the material’s ability to retain water, adsorb nutrients, exchange gases, and support microbial colonization. Additionally, well-developed pore networks increase carbonaceous materials’ potential for adsorption and help to improve soil quality over time [46]. Beyond these, as indicated in Fig.2, the charcoal retained a significant amount of OC (47.6 ± 2.38%). This OC content is lower than the OC content of leaves of *Cordia africana*, which values varied from 62.2% to 63% [50]*.* The OC content of charcoal could have a significant role as a soil amendment for improving soil fertility and carbon sequestration [53]. The volatile organic matter content was 18.5 ± 0.92%, which is lower than the reported values for agricultural-residue biochars and suggests effective carbonization during production. The lower volatile matter might be due to the increased pyrolysis temperature, which gradually removed it and formation of greater aromatic carbon. Biochars with lower volatile organic matter content are also more suitable for long-term environmental applications and carbon sequesteration [46].

### 3.2 Physico-chemical properties of ACDB

**Fig 3:**
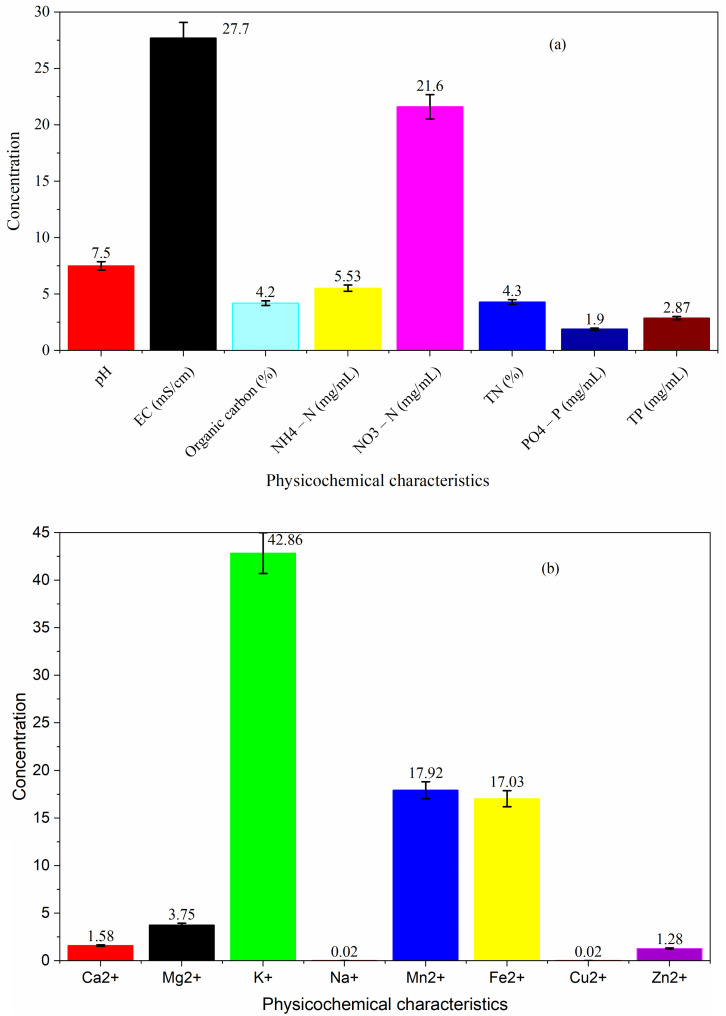
ACDB properties. (a) pH, EC, OC, nutrients; (b) exchangeable cations and trace elements.

Analysis of the physico-chemical characteristics of ACDB is indicated in Fig 3(a) (b). The mean pH of ACDB was 7.5 ± 0.38, which is somewhat neutral. Microbial breakdown of organic matter during anaerobic digestion produces bicarbonates and ammonium compounds, which tend to raise pH and stable the digestate near neutral to slightly alkaline conditions. Depending on the content of the feedstock and the conditions of digestion, digestate pH values typically fall between 7.0 and 8.8 [54]. This pH value met the FAO pH standard, which varied from 6.5 to 8.4, recommended for agricultural reuse purposes. When applied to acidic agricultural soils, a slightly alkaline pH may assist reduce soil acidity and enhance nutrient availability [54]. Prior findings showed that lower pH (< 6.5) promotes leaching of exchangeable cations, while higher pH (>11) causes soil microorganisms’ death and inactive movement of ions, resulting in nutritional imbalance and plant growth restriction [55]. The bioslurry’s EC of 27.7 ± 1.38 mScm ⁻ ¹ (Fig 3(a)) indicated a comparatively high soluble ion concentration. This value falls again within the FAO standard limit range of 0–3 dS.m^-1^ and indicating the presence of mineral ions that influences soil nutrient availability and plant growth. On the other hand, its large rate application may cause soil structure clumping and reduce the absorptivity of soil, and may also result in soil and plant toxicity, depending on the initial soil nature [55], and also affects tomato plant growth, water transport, and nutrient uptake potential [56]. In order to optimize agronomic benefits and minimize potential salt stress, treatment rates should be carefully controlled [57].

The OC and nutrient content analysis in ACDB reveal promising results (Fig 3(a)). The residual OC content was 4.2 ± 0.21%, which is relatively lower; indicating the most organic matters are degraded by bacteria and converted into carbon dioxide and methane, which reduce its amount in the digestate. However, land application of this residual carbon fraction can enhances soil organic matter and promotes microbial activity. Digestates contain significant amounts of stabilized organic matter that can improve soil structure, water retention, and nutrient-holding capacity [58]. On the contrary, relatively higher TN and TP values were found in the ACDB, with respective values of 4.3% and 2.87 mgL^-1^ (Fig 3 (a)), which may be due to the mineralization of nutrients from complex organic matter through the anaerobic digestion process. The bioslurry is a useful source of nitrogen for crop productivity, as evidenced by its total nitrogen level of 4.3 ± 0.22%. This study’s TN is preserved during digestion, which raises the bioslurry’s fertilizer value and reduce the need for artificial nitrogen fertilizers [59]. The bioslurry has also contained significant phosphorus content (2.87 ± 0.14 mgL ⁻ ¹) (Fig 3 (a)). This higher content may be due to its less fractionation into gaseous compounds during anaerobic digestion; it is not substantially lost like carbon and nitrogen. As a result, digestates frequently keep the majority of the phosphorus that was initially present in the raw materials. Phosphorus increases the fertilizer value of bioslurry and promotes crop productivity, root development, and energy transfer. Additionally, some of the phosphorus transformed into more soluble forms via anaerobic digestion, making it more accessible to plants [54]. The bioslurry also contains direct plant available nitrogen species such as NH_4_ – N and NO_3_ – N with respective values of 5.53 ± 0.28 mgL^-1^ and 21.6 ± 0.28 mgL^-1^ (Fig 3(a)). This significant nitrogen mineralization during digestion is the predominant inorganic nitrogen component in many anaerobic digestates. Because ammonium is easily taken by plants, is a crucial indicator of digestate fertilizer quality [59]. NO_3_ – N is often restricted in completely anaerobic environments, but it is a highly plant-available nutrient. Furthermore, the presence of easily accessible phosphorus in the bioslurry is further indicated by the PO_4_ – P concentration of 1.9 mg mL ⁻ ¹. The PO_4_^3-^ contributing to enhancing plant productivity through playing a significant role in energy transfer, photosynthesis, and root development [60]. However, excessive application of nutrient-rich ACDB may cause toxicity and laxative effects on crop yield and can delay ripening and maturity [61].

The ACDB also contains promising exchangeable cations such as Ca^2+^, Mg^2+^, Na^+^, and K^+^ (Fig 3(b)). The presence of Ca^2+^ and Mg^2+^ in ACDB is important for improving soil fertility, plant growth, and crop yield [62]. Na^+^ is also important for plant growth, but its excess presence causes soil salinization that can promote osmotic stress in plants to absorb water and nutrients [63]. K^+^ is an essentially required plant nutrient which can regulate enzyme activation, water uptake, and plant health. The determined value of K^+^ in ACDB is 42.86 mgL^-1^, which can contribute to optimal plant growth and yield. In this study, the exchangeable cation concentrations were less than the European Catchment Management Agencies’ recommended standards of 150 mgL^-1^, 12 mgL^-1^, 50 mgL^-1^, and 50 mgL^-1^, respectively, for Na^+^, K^+^, Mg^2+^, and Ca^2+^, which do not cause substantial effects on crop production and soil physico-chemical properties [63,64]. The presence of trace elements in ACDB can be used as a micronutrient supplement for plant growth. As indicated in Fig 3(b), the Mn^2+^, Fe^2+^, Cu^2+^, and Zn^2+^ values were 117.92 mgL^-1^, 17.03 mgL^-1^, 0.02 mgL^-1^, and 1.28 mgL^-1^, respectively, which are much lower than the recommended concentrations, and can be used for agricultural application in environmental friendly approach with no toxicity [38]. Overall, the ACDB’s physicochemical characteristics indicates a potential promise as an organic fertilizer, which can enhance soil fertility and promote crop growth because of its almost neutral pH, high nutrient concentrations, and significant amounts of plant-available macro- and micronutrients.

### 3.3 Physico-chemical characteristics of ACDBCFs

The analyses of the physico-chemical properties of ACDBCFs are indicated in Table 3. As indicated in the Table, the pH is alkaline, which varied from 8.9 to 9.63. The alkaline properties of ACDBCFs may be due to the presence of higher concentrations of exchangeable cations, which can help to reduce soil acidity by releasing basic cations and enhance plant growth [65,66]. However, a higher pH values of 9.6 and 9.5 were obtained in ACDBCF5 and ACDBCF4, respectively, may be due to addition of 10% and 5% charcoal, which can adhere higher concentrations of base cations, and can potentially releases them into the soil solution that helps to reduce acidity in the soil through nutrients adsorption and enhances good crops seed germination and growth [66]. The EC analysis of ACDBFCs result also fluctuated from 725.5 to 1343 mScm^-1^ (Table 3). These EC values can be relevant for agricultural applications due to their ion exchange capacity [30]. The ACDBCF4 had a relatively higher value (1343 mScm^-1^), followed by ACDBCF5 (1139 mScm^-1^), obtained under the addition of 5% and 10% charcoal, and can be relevant for agricultural applications due to their higher exchangeable cations [30].

**Table 3:** Physico-chemical characteristics of ACDBCFs.

ACDBCF types	Physicochemical parameters											
	pH	EC (mScm^-1^)	OC (%)	NH_4_ – N (mgL^-1^)	NO_3_ – N (mgL^-1^)	TN (%)	PO_4_ – P (mgL^-1^)	TP (mgL^-1)^	Na^+^ (mgL^-1)^	K^+^ (mgL^-1^)	Mg^2+^ (mgL^-1^)	Ca^2+^ (mgL^-1^)
ACDBCF1	8.9 ± 0.0	984.7 ± 0.01	35.2 ± 5.60	2.5 ± 1.40	11.9 ± 4.50	5.2 ± 2.10	0.74 ± 0.0	17.4 ± 1.80	4.45 ± 0.0	49.4 ± 5..80	1.88 ± 0.0	28.7 ± 0.04
ACDBCF2	9.4 ± 0.0	1008 ± 0.02	26.8 ± 5.20	2.8 ± 0.80	12.2 ± 4.30	4.7 ± 1.20	1.4 ± 3.50	16.5 ± 1.50	9.2 ± 0.01	44.5 ± 1.60	1.8 ± 1.82	36.2 ± 0.04
ACDBCF3	9.3 ± 0.0	725.5 ± 0.03	45.2 ± 7.90	3.05 ± 0.0	14.8 ± 4.70	5.3 ± 0.50	2.8 ± 0.30	14.8 ± 4.90	4.81 ± 0.0	49.4 ± 5.10	2.1 ± 0.96	99.9 ± 0.01
ACDBCF4	9.5 ± 0.0	1343 ± 0.01	54.2 ± 7.20	4.2 ± 1.2 0	17.6 ± 4.50	9.6 ± 0.50	2.1 ± 2.30	15.1 ± 5.10	5.1 ± 0.01	49.9 ± 5.07	2.2 ± 1.0	125 ± 0.01
ACDBCF5	9.6 ± 0.0	1139 ± 0.04	65.6 ± 5.40	4.6 ± 0.60	18.2 ± 4.10	9.9 ± 0.20	3.1 ± 0.70	18.2 ± 1.80	10.12 ± 0.0	55.7 ± 5.70	2.4 ± 0.40	155.5 ± 0.30

The value of the OC content was varied from 26.83 to 65.62% (Table 3). The application of 5% and 10%charcoal enhances the OC content of ACDBCFs to the range of 54.2 to 65.62%. These might be due to the adsorption of labile carbon contents [66] and the additive effect of charcoals. Hence, the application of ACDBCFs as soil amendments can significantly impact the soil’s organic fertility. In general, the higher OC contributes to soil fertility and release nutrient for plant growth [65]. TN content was varied from 5.35 to 9.97%. The highest amount of TN (9.9%) was observed in ACDBCF5 under the addition of 10% charcoal into the ACDB, followed by ACDBCF4 under the addition of a 5% charcoal into the ACDB. On the other hand, the NH_4_ – N and NO_3_ – N were fluctuated from 2.5 to 4.6 mgL^-1^ and 11.9 to 17.6 mgL^-1^, respectively. The relative maximum available NH_4_ – N and NO_3_ – N were observed in ACDBCF5 (4.6 mgL^-1^ and 18.2 mgL^-1^) followed by ACDBCF4 (4.2 mgL^-1^ and 17.6 mgL^-1^), respectively. Several studies have been also reported the significant increase in soil OC and TN contents upon subsequent mixing of biochar with organic fertilizers [22]. TP and PO_4_ – P are also the most important elements for growth, cell division, root growth and elongation, seed and fruit development, and early ripening. Furthermore, they also help in energy storage and transfer [65]. The TP and PO_4_ – P values were ranged from 17.4 to 18.23 mgL^-1^ and 0.74 to 3.13 mgL^-1^, respectively (Table 3). This phosphorus availability can plays for proper plant growth, and enhances the availability of phosphorus in the soil. Furthermore, the ACDBCFs have also contained other essential nutrients such as K^+^, Ca^2+^, Mg^2+^, and Na^+^ (Table 3). K^+^ plays an important role in different physiological processes of plants. K^+^ is a major nutrient for the production of superior quality crops, and plays a catalytic role in nature. The analysis of K^+^ in ACDBCFs varied between 44.5 and 55.7 mgL^-1^. The ACDBCFs Ca^2+^, Mg^2+^, and Na^+^ values also varied in between 28.75 to 155.55 mgL^-1^, 1.77 to 2.43 mgL^-1^, and 4.45 to 10.12 mgL^-1^, respectively (Table 3). This suggests that these exchangeable cations can provide a rich source of micro-nutrients for plants.

### 3.4 Effect of ACDBCFs amendment on tomato agronomic data

The application of ACDBCFs significantly influenced tomato growth (leaf number, plant height, and stem diameter) and yield parameters (fruit yield and dry biomass yield) compared with the control (Table 4). The obtained data for the number of leaves fluctuated from 5.67 to 10.67 under the different treatment groups. As indicated in Table 4, the highest leaf number (10.67 leaves plant ⁻ ^1^) was recorded under T8, followed by T5 (10.00 leaves plant ⁻ ^1^) and T6 (9.67 leaves plant ⁻ ^1^), whereas the lowest value (5.67 leaves plant ⁻ ^1^) was observed in the control treatment (T1). The ANOVA (Turkey test) analysis between these treatments revealed that there is insignificant variation (P = 0.9994 for T5 and T6, P = 0.81051 for T6 and T8, and P = 0.97935 for T5 and T8 (Table 4). Furthermore, leaf number was also showed insignificant variation with between treatment groups of T3, T4, and T7. The relatively higher leaf number counts obtained under all the treatment groups except T1 and T2 may be attributed to the enhanced supply of essential nutrients, particularly NPSB and ACDBCFs, which enhances the growth of the local tomato variety. Nitrogen plays a crucial role in chlorophyll formation and vegetative growth, thereby promoting leaf initiation and expansion. Similar findings have been reported by Agegnehu et al. [22], who observed increased leaf production in crops amended with biochar-based organic fertilizers due to improved nutrient retention and nutrient-use efficiency. Additionally, Rehman & Razzaq [67] reported a significantly higher number of tomato growth parameters amended with biochar with farmyard manure under optimal mineral fertilizing due to the availability of important plant nutrients.

**Table 4:** Effects of ACDBCFs amendment on tomato biometric data.

Treatment types	Tomato biometric data measurements				
	Leaf number (Leaves plant ⁻ ¹)	Plant height (cm)	Stem diameter (cm)	Fruit yield (kgpot^-1^)	Dry biomass yield (kgpot^-1^)
T1	5.67^c^	7.00^c^	1.83^b^	0.71^d^	0.05^b^
T2	7.67^bc^	13.91^abc^	1.87^b^	2.2^b^	0.05^b^
T3	8.67^ab^	13.66^abc^	2.10^ab^	2.60^ab^	0.06^b^
T4	8.55^ab^	14.51^abc^	2.03^ab^	2.63^ab^	0.07^b^
T5	10.00^ab^	17.66^abc^	2.40^ab^	2.67^a^	0.07^b^
T6	9.67^ab^	15.50^ab^	2.37^ab^	2.87^a^	0.10^a^
T7	8.33^ab^	13.50^bc^	2.27^ab^	1.63^c^	0.06^b^
T8	10.67^a^	22.00^a^	2.57^a^	2.73^a^	0.09^a^
CV (%)	9.73	2.03	9.01	6.79	14.24
P – value	<0.0001	<0.0014	<0.004	<0.0001	<0.0001

CV = Coefficient of variation, different superscript letters indicate significant variation (at p < 0.05)

Plant height was also significantly affected by NPSB inorganic fertilizer and ACDBCFs application, with the greatest plants recorded under T8 (22.00 cm), followed by T5 (17.66 cm) and T6 (15.50 cm). The lowest plants height was observed in the control treatment (7.00 cm). The ANOVA analysis indicated that treatment groups showed significant variations (p < *0.0014*) from each other. However, the Turkey post hoc test analysis revealed a clear variation between treatment groups T7 and T8 (p = 0.04648), T1 and T8 (2.874E-4), T1 and T6 (0.04893), and T1 and T5 (p = 0.00875) (Table 4). The relatively higher tomato plant height was attained by T5 and T6 as compared to all other ACDBCFs treatments, indicates that the application ACDB + charcoal (+5%), and ACDB + charcoal (+10%) were contributed positive impact on the plant height of tomato. Furthermore, other ACDBCFs amended pot soils were also relatively increased in tomato plant height, which might be due to the role of charcoal to improve soil physicochemical properties, such as increasing soil pore space, surface area, increasing soil pH, and nutrient availability [49], while bioslurry supplies readily available nutrients. Similar improvements in tomato plant height have been reported by Lehmann and Joseph [15], who observed that application of biochar-compost enhance nutrient availability and improve soil structure as major drivers of increased vegetative growth. Additionally, Simiele et al. [68], also reported that that the BBOFs enhance tomato plant height growth and yield due to the better availability of soil nutrients that enhance the growth of plants by increasing cell division and elongation. This more closely associated with improved soil chemical properties, such as pH, cation exchange capacity, organic matter content, and nutrient availability to sustain plant growth and optimize crop yield [49].

Tomato stem diameter measurement results are shown in Table 4. As indicated in the Table, the stem diameters per tomato plant ranged from 1.87 to 2.57 cm. T1 has a significantly lower stem diameter (1.83 cm) compared to all other treatments; while T8, followed by T5 and T6, showed the highest stem diameter. Although differences between treatment treatments were statistically similar, the trend showed relatively more thicker stems in ACDBCF- particularly in ACDB + charcoal (+5%), and ACDB + charcoal (+10%) amended pots. The observed increased performance response of tomato plants’ stem diameter in these treatments may be associated with the improved nutrient acquisition and longer time availability of a sufficient supply of nutrients, and reduced contents of soluble salts, which support stronger structural development. The enhanced stem growth observed in this study may also reflect improved soil moisture retention provided by charcoal, which reduces water stress and promotes continuous plant growth. In argument with the present study findings, Murtaza et al. [69] reported a significantly increased stem diameter of the tomato plants under biochar addition due to the removal of toxic nutrients, which could have limited the plant growth. In contrast, higher fruit yield was obtained from T6 (2.87 kg pot ⁻ ¹), followed closely by T8 (2.73 kg pot ⁻ ¹), and T5 (2.67 kg pot ⁻ ¹) (Table 4). Whereas, the control treatment produced the lowest yield of 0.70 kgpot ⁻ ¹. The Duun – Sindak post hoc test revealed that majority of the treatment groups have significant variations except some few examples such as T6 and T8 (p = 0.99996), and T5 and T6 (p = 0.97925) and others indicated in Table 4. The equivalent higher fruit yield productivity, particularly found in the T6 and T5 treatments, might be closely associated with the effectiveness of ACDB + charcoal (+10%) and ACDB + charcoal (+5%) amendments, which supply essential nutrients throughout the growing period, in which the bioslurry provides readily available nitrogen, potassium, and phosphorus, while charcoal enhances nutrient retention and minimizes nutrient losses through leaching. Consequently, nutrient availability is maintained for longer periods, resulting in improved soil chemical properties (such as pH, cation exchange capacity, organic matter content, and nutrient availability), and sustain plant growth and fruit development. [49]. In argument to this study, Murtaza et al. [69] reported that the biochar addition as soil amendment increased the tomato plant growth and yield components due to improved soil organic matter, and essential nutrients to plants, and increased nutrient use efficiency to facilitate better plant growth and yield. Furthermore, previous study evidences have also reported similar yield increases in tomato plant due to the applications of biochar-enriched organic fertilizers, contributing to improved nutrient cycling, soil microbial activity, and water-use efficiency [15,22]. Dry biomass yield (DBY) was brought a similar pattern to fruit yield (Table 4). The highest DBY (0.10 kgpot ⁻ ¹) was recorded under T6 and T8, whereas the lowest DBY (0.05 kg pot ⁻ ¹) were occurred in T1 and T2. The relatively higher and significantly varied DBY observed under T6 and T8 indicates that application of ACDB + charcoal (+10%) and inorganic fertilizer performs most effectively in promoting dry biomass production, which might be due to enhanced photosynthetic efficiency and nutrient utilization. Especially, the good pore volume of charcoal may contributed to improved soil moisture storage and sufficient nutrients to sustain plant growth. According to Lehmann and Joseph [15], BBOFs often increase biomass production through improvements in soil physico-chemical and biological properties. The observed improvements in tomato growth and yield parameters highlight the combined interaction between bioslurry and charcoal co – composted fertilizers. Besides, other ACDBCF treatment groups have also contributed a positive effect on the DBY of the tomato plant. This study result is in argument with the Amin et al. [49] reported result that the application of BBOFs improved the total plant biomass weight of crops.

**Fig 4:**
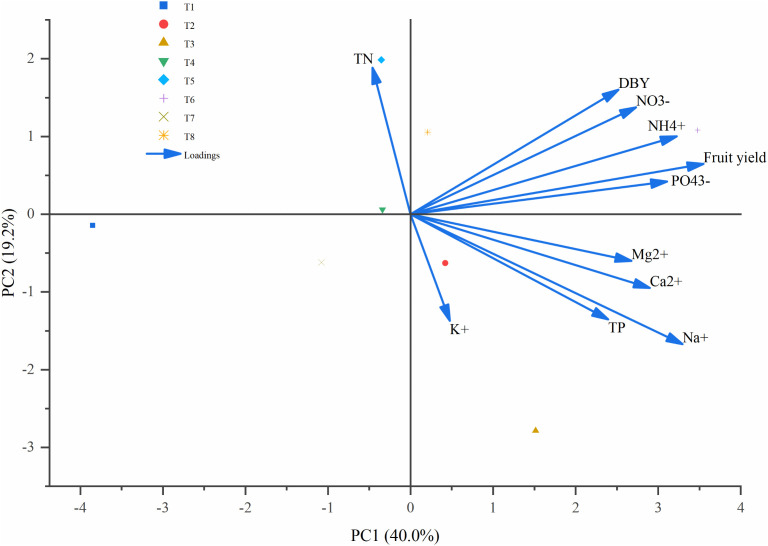
PCA biplot illustrating the dimensional relationship between ACDBCF treatments and tomato productivity.

In addition to the ANOVA results, the dimensional association of amendments and tomato productivity was observed. The PCA also accounted a clear dimensional relationship about the effects of amendments on the tomato productivity. As indicated in Fig 4, the PCA2 direction accounted for 19.2% of the variation, while the PCA1 dimension accounted 40.0% of the variation, in which the PCA1 direction showed a strong correlation between the tomato yield attributes (fruit yield and DBY) and amendments. Among the treatments, treatments like T2, T3, T5, T6, and T8 were positioned along the positive side of PC1, mostly characterized by enhanced productivity. This positive response of tomato productivity is mainly associated with the availability of nitrogen and phosphorus, which are the main influencing factor for tomato productivity. For example, nitrogen is essential for chlorophyll production, protein synthesis, and enzymatic activity, which has a direct impact on photosynthetic efficiency and biomass accumulation [70]. In line with this, previous studies also showed that application of sufficient nitrogen sources improve vegetative growth, photosynthetic capability, and yield in tomato crops through promoting chlorophyll, easily available input of NH₄⁺ and NO₃ ⁻ , amino acids, nutrient usage efficiency, and enzymes [70–72]. Additionally, phosphorus can also made a significant contribution by supporting reproductive development and energy transmission, especially throughout the periods of flowering and fruit formation. According to Havlin et al. [71], phosphorus availability enhances tomato flower formation and fruit yields. The essential role of phosphorus in reproductive development and energy transmission, especially during blooming and fruit yield stages is further enhanced by the presence of orthophosphate availability [72]. Conversely, treatments like T1, T4, and T7 were situated away from the fruit and DBY yield vectors and closer to the PCA2 axis, showed low correlations. This weak association may be due to the availability of insufficient nutrients, which reduces the crop yield productivity [70]. The lower-right quadrant of the biplot represented the cationic nutrients Na ⁺ , Ca² ⁺ , and Mg² ⁺ , which also had a good correlation with tomato yields. Although Ca²⁺and Mg² ⁺ are necessary for the production of chlorophyll and the stability of cell walls, respectively, their contribution to yield is frequently indirect and depends on sufficient nitrogen availability [71].

### 3.5 Effect of ACDBCFs amendment on soil physico-chemical properties

**Fig 5:**
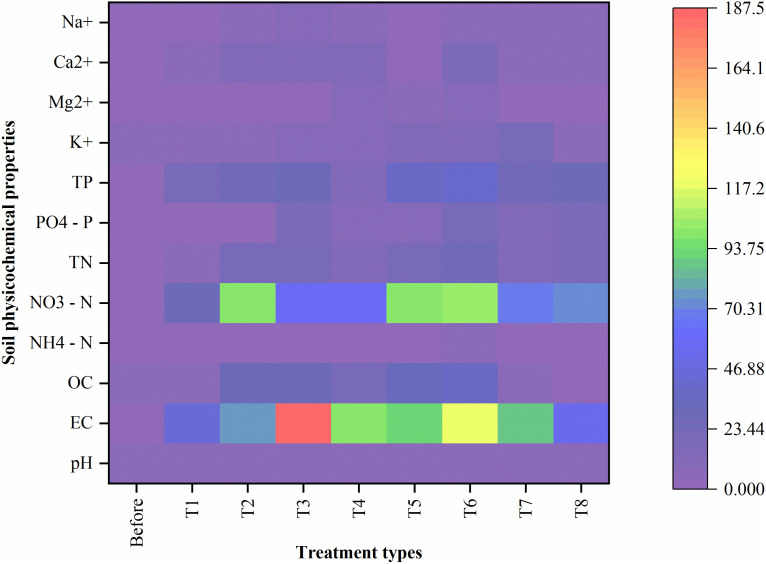
Heat map visualization of effects of soil amendments on the background soil properties.

The amendments of ACDBCFs significantly affected some soil physico-chemical characteristics compared with the initial and control soil conditions (Table 5). For each treatment group, the background soil pH was 6.43, indicating slightly acidic conditions. Following different treatment applications, soil pH ranged from 6.16 (T1) to 7.05 (T6). The significantly higher pH increments (Table 5) were observed under the amendments of ACDB + charcoal (+10%) and ACDB + charcoal (+5%), respectively. This indicates that ACDBCFs contributed to reducing soil acidity and providing a more neutral soil conditions, leading to meet the optimal pH range of 6.5 to 7.5, ideal for plant growth via readily available nutrient uptake [73]. The reasons for this pH increase may be attributed to the alkaline nature of charcoal (Fig 2) and ACDB (Fig 3(a)), which can neutralize soil acidity and enhance nutrient availability. Furthermore, this increase in pH may be attributed to the synergistic effects of charcoal and ACDB, which enhance the rapid release of exchangeable cations into the soil solution, thereby decreasing acid-causing substances such as H^+^ and Al^+3^ [74]. Other similar study findings have been also reported by Liu et al. [75] and Lehmann and Joseph [15], who reported that the application of biochar- co-mixed with composts improved the acidic nature of soils towards alkaline nature due to the presence of increased basic cations. Similarly, the application of ACDBCFs also significantly increased the soil EC from its background value of 2.80 mScm^-1^ to values varied between 77.20 and 187.10 mScm^-1^ (Table 5). This markedly increment under the ACDBCF’s application suggests presence of exchangeable cations and anions that can increase soil pH and EC [76]. In argument to this study, Antonangelo et al. [76,77] reported that application of BBOFs significantly increases soil EC, due to the presence of more exchangeable cations and anions. Furthermore, Agegnehu et al. [2] also reported that compost and biochar applications increased EC due to the release of mineral nutrients. In line with this statistical analysis, the heat map visualization in Fig 5 also indicates that most treatments exhibit positive correlations, and thus reflect substantial enrichment of soil physico-chemical constituents due to the addition of ACDBCFs and ACDB applications. Despite a slight increase in pH, there is comparatively greater EC variance between treatments, indicating that ACDBCFs can change acidity/alkalinity levels.

**Table 5:** Effect of ACDBCFs application on soil physico-chemical characteristics.

Treatment types	Physico-chemical parameters											
	pH	EC (mScm^-1^)	OC (%)	NH_4_ – N (mgL^-1^)	NO_3_ – N (mgL^-1^)	TN (%)	PO_4_ – P (mgL^-1^)	TP (mgL^-1^)	K^+^ (mgL^-1^)	Mg^2+^ (mgL^-1^)	Ca^2+^ (mgL^-1^)	Na^+^ (mgL^-1^)
Before	6.43^f^	2.75^i^	4.38^g^	1.53^g^	2.56^i^	1.29^i^	0.57^i^	0.94^i^	4.86^f^	3.75^e^	1.57^h^	0.02^i^
T1	6.18 ^g^	43.18^h^	4.20^h^	0.87 ^i^	27.^78h^	6.14^h^	2.72 ^h^	19.90^f^	4.83^f^	1.31^i^	4.50^f^	2.20^h^
T2	6.72 ^e^	77.25^f^	28.20^d^	1.8 ^e^	98.50 ^c^	22.3^0c^	3.43^g^	25.3^6e^	4.80 ^f^	1.79^h^	12.9 ^c^	5.89^c^
T3	6.75^e^	187.2^a^	28.55^c^	1.77^f^	55.0^g^	21.30 ^d^	16.78^c^	29.74^c^	10.90 ^d^	3.78^e^	12.89^c^	10.30^a^
T4	6.83^d^	100.05^c^	23.14^e^	1.92^d^	56.^05f^	12.0^g^	9.87^f^	15.10^h^	9.80^e^	11.50^a^	14.50^b^	5.80^e^
T5	6.96^b^	92.05^d^	33.50^b^	2.55^b^	99.60 ^b^	22.80^b^	11.30 ^e^	36.30^b^	13.50^c^	6.32^c^	3.65^g^	3.69^g^
T6	7.05 ^a^	118.65^b^	37.50 ^a^	3.92^a^	102.0 ^a^	24.10^a^	22.80^a^	39.90^a^	14.70^b^	10.50^b^	15.90^a^	7.39^b^
T7	6.89^c^	89.19^e^	5.70^f^	2.03^c^	69.0^e^	15.23^f^	12.40^d^	25.24^f^	20.30^a^	2.88^f^	5.12^d^	5.40^d^
T8	6.39^f^	53.28 ^g^	3.0^i^	1.26^h^	73.0^d^	15.84^e^	18.50^b^	28.62^d^	4.30^i^	2.32^g^	4.82^e^	5.84^e^
CV (%)	17	8.14	13	17.3	17.3	2.25	4.84	6.16	6.39	6.91	6.28	1.05
P – value	<0.0001	<0.0001	<0.0001	<0.0001	<0.0001	<0.0001	<0.0001	<0.0001	<0.0001	<0.0001	<0.0001	<0.0001

CV = Coefficient of variation, different superscript letters in columns indicate significant variation (at p<0.05)

Analysis of soil OC and nutrient contents showed considerable improvements following ACDBCFs application (Table 5). The background soil OC content was 4.38%, but after ACDBCFs application, it fluctuated from 23.14 to 37.51% (Table 5). The maximum increment was observed under treatment T5 (33.5%) and T6 (37.51%) with the application of higher proportions of bioslurry and charcoal (i.e., ACDB + charcoal (+10%) and ACDB + charcoal (+5%)).On the other hand, the control and NPSB treated pot soils showed slightly lower results from the background soil OC content. The promising soil OC outcome as a result of ACDBCFs is in agreement with the previous study evidence of the use of BBOFs improving soil organic matter content due to their carbon-rich natures [78]. The presence of OC in BBOFs contributed to an improved soil organic matter content and microbial activity [79]. Furthermore, application of biochar-enriched fertilizers are essential for the formation and stabilization of soil structure, higher water-holding capacity, retention of nutrients, and contributes to long-term carbon storage and improved soil quality [15,80]. Besides this soil OC improvement, the application of ACDBCFs also showed an encouraging shift in soil nutrient contents (Table 5). The background TN and TP content of treatment pot soil were 1.29% and 0.94 mgL^-1^. The highest TN and TP concentrations were observed in T6 (24.10% and 39.9 mgL^-1^), followed by T5 (22.80% and 36.3 mgL^-1^) (Table 5). The significantly varied maximum change of TN and TP was observed in all ACDBCFs amended pot soils, particularly under the higher charcoal added bioslurry treatment groups, which boost essential nutrients in the soil and are more important for crop growth and productivity. The increase of soil TN and TP can be assigned to the relatively higher nutrient availability in the ACDB waste [79], and might also in the charcoal waste which can contribute synergetic effect on the soil nutrients retentions. In argument with this study, a study report indicated that the use of BBOFs improves soil TN and TP contents [78]. The content of available nutrients such as NH_4_ – N, NO_3_ – N, and PO_4_ – P were also showed a significant increment under the application of ACDBCFs (Table 5). From the treatment groups, the highest NH_4_–N, NO_3_ –N, and PO_4_ – P concentrations were recorded in T6 with respective values of 3.92, 102.0, and 22.8 mgL^-1^ (Table 5). This suggests that the application of charcoal co-composted with ACDB can play a significant role in soil available nutrients might be due to its synchronization in the charcoal or biochar surface entrapment of nutrients by surface functional groups [81]. This finding is in agreement with Singh et al. [76], who report that biochar amendments enhanced available phosphorus retention and availability in soils. Similarly, Phillips et al. [78] suggest that biochar additions increased soil nitrogen content through enhanced nutrient retention and reduced nitrogen losses. Agegnehu et al. [2] have been also reported that biochar –compost mixtures improve phosphorus retention and availability in soils. Besides, the heat map visualization also shows that ACDBCF treatments significantly raised soil nutrient and OC concentrations (Fig 5). Strong colors shown in the background region indicates that OC has significant increases in T2, T3, T5, T6, and T7, which is essential for increased nutrient cycling and microbial activity [82]. Additionally, the nutrient heat map display shows significant enrichment particularly in T2, T5, and T6, with nitrogen species. According to the heat map, NO_3_ - N and TN exhibit larger positive deviations than ammonium (NH_4_ - N) in the majority of treatments, which might be due to higher microbial activity, better aeration, or more organic matter availability [82]. Throughout T3–T8, PO_4_^3^⁻ and TP both exhibit consistently high scores, with T6 and T5 showing the highest responses. This slight increase in PO_4_^3^⁻ and TP indicates increased phosphorus mobilization as a result of synchronization potential of the biochar adsorbent [83].

Moreover, analysis of the residual effects of ACDBCFs on soil exchangeable cations, such as Ca^2+,^ Mg^2+^, K^+^, and Na^+^ revealed significant changes as compared to other treatments (Table 5). As indicated in Table 5, the background soil exchangeable cations increased due to the combined effect of both charcoal and ACDB fertilizer. The relatively higher K⁺ concentration was observed in T7 (20.30 mgL^-1^), followed by T6 (14.70 mgL^-1^) and T5 (13.50 mgL^-1^). This soil K⁺ enrichment may arises from the ACDB and ash fraction of charcoal. K ⁺ is crucial for plant growth, water regulation and enzyme activation. Mg^2+^ concentrations varied significantly among treatments. The highest value was recorded in T4 (11.50 mgL^-1^) followed by T6 (10.50 mgL^-1^. Ca^2+^ concentrations significantly varied between treatment groups with the highest value obtained under T6 (15.90 mgL^-1^), followed by T4 (14.50 mgL^-1^). This increased Ca^2+^ availability contributes to improved soil nutrient balance. Na^+^ concentrations also showed improvements from the background conditions (Table 5). Although Na^+^ levels increased significantly, the recorded concentrations are considerably found below the soil salinity threshold limit. Among treatments, T6 consistently resulted significant changes in soil physico-chemical characteristics, which suggests that ACDB + charcoal (+10%) use is effective for soil fertility improvement. Similar study report indicated that the use of BBOFs improves soil exchangeable cations content, which might be due to their carbon-rich nature [84]. The presence of exchangeable cations in BBOFs contributed to an improved soil cation exchange capacity and microbial activity [79]. Heat map responses also indicated that K⁺ and Ca² ⁺ exhibit significant positive shifts, while Mg²⁺ and Na⁺ show more moderate but still positive shifts.

## Conclusion

Characterization of the physico-chemical properties of charcoal waste revealed high-quality characteristics in terms of pH, pore volume, bulk density, OC, and EC. Analogously, characterization of the ACDB also revealed promising physico-chemical properties. Hence, formulation of ACDBCFs from both charcoal and ACDB brought promising macro- and micro-nutrients, which can serve as effective amendments for enhancing the soil fertility, particularly on soil acidity, and reduces nutrients losses and facilitate crop productivity. The pot experimental study indicated that the application of ACDBCFs significantly improved the tomato biometric measurement data, and the acidic soil properties such as soil pH, EC, TN, TP, OC, nutrients, and exchangeable cations (Ca^2+^, Mg^2+^, K^+^, and Na^+^). PCA result also reveals a strong association between nutrient availability, fruit, and biomass yields of tomato. The heat map visualization further confirms that application of ACDBCFs significantly improved background soil nutrients. Overall, a finding from this short-term pot experimental investigation, application of ACDBCFs enhances tomato productivity and soil quality. We recommend researchers to carryout long-term monitoring at field level validation in representation of real soil conditions and nutrient dynamics to promote resource recovery, thereby supporting national socioeconomic and environmental sustainable development.

## Supporting information

S1 File Tomato biometric data and ANOVA. (PDF)
