# Supplementary material for: Synergetic effect of bioslurry and charcoal co – composted fertilizers on soil properties and tomato (Solanum lycopersicum) productivity
Source: PLoS One. 2026 Jul 28;21(7):e0353951. doi: 10.1371/journal.pone.0353951 (PMC13411927; doi:10.1371/journal.pone.0353951)
Supplement: S1 File — (PDF) [file pone.0353951.s001.pdf]

[S1. Tomato biometric data and ANOVA]

[S1 Table. Tomato biometric data]

Treatments	Leaf number	Plant height	Stem diameter	Fruit Yield	Dry BMY
T1	5.56	6	2	0.72	0.35
T1	5.67	7	1.8	0.74	0.3
T1	5.74	8	1.8	0.68	0.35
T2	8.11	11.72	2.1	2.4	2.1
T2	7.67	14	1.87	2.7	1.87
T2	7.24	16	1.65	2.8	1.65
T3	7.44	11.72	2.4	2	0.35
T3	8.67	13.67	2.1	2.3	0.5
T3	9.89	15.58	1.8	2.4	0.4
T4	8	12.44	2.2	2.5	0.5
T4	8.64	14.5	2.2	2.9	0.5
T4	9	16.6	1.7	2.5	0.35
T5	9.11	12	2.27	2.5	0.5
T5	10	17.67	2.4	2.7	0.4
T5	11	23.3	2.52	2.8	0.6
T6	8.33	12.33	2.25	2.7	0.6
T6	9.67	15.5	2.37	2.8	0.6
T6	10.97	18.6	2.5	3.1	0.6
T7	7.8	12.78	2.13	1.5	0.45
T7	8.33	13.5	2.27	1.6	0.4
T7	8.89	14.2	2.4	1.8	0.5
T8	9.78	18.11	2.6	2.6	0.4
T8	10.67	22	2.7	2.7	0.6
T8	11.56	26	2.4	2.9	0.5

[S2 Table. ANOVA for Leaf number]

		MeanDiff	SEM	q Value	Prob	Alpha	Sig	LCL	UCL
T2	T1	2.01667	0.68802	4.14525	0.13038	0.05	0	-0.36535	4.39868
T3	T1	3.01	0.68802	6.18704	0.00862	0.05	1	0.62799	5.39201
T3	T2	0.99333	0.68802	2.04179	0.82453	0.05	0	-1.38868	3.37535
T4	T1	2.89	0.68802	5.94038	0.01211	0.05	1	0.50799	5.27201
T4	T2	0.87333	0.68802	1.79513	0.89744	0.05	0	-1.50868	3.25535
T4	T3	-0.12	0.68802	0.24666	1	0.05	0	-2.50201	2.26201
T5	T1	4.38	0.68802	9.00306	1.99E-04	0.05	1	1.99799	6.76201
T5	T2	2.36333	0.68802	4.85782	0.0526	0.05	0	-0.01868	4.74535
T5	T3	1.37	0.68802	2.81603	0.51578	0.05	0	-1.01201	3.75201
T5	T4	1.49	0.68802	3.06269	0.41835	0.05	0	-0.89201	3.87201
T6	T1	4	0.68802	8.22198	5.46E-04	0.05	1	1.61799	6.38201
T6	T2	1.98333	0.68802	4.07673	0.14165	0.05	0	-0.39868	4.36535
T6	T3	0.99	0.68802	2.03494	0.82682	0.05	0	-1.39201	3.37201
T6	T4	1.11	0.68802	2.2816	0.73675	0.05	0	-1.27201	3.49201
T6	T5	-0.38	0.68802	0.78109	0.99904	0.05	0	-2.76201	2.00201
T7	T1	2.68333	0.68802	5.51557	0.0217	0.05	1	0.30132	5.06535
T7	T2	0.66667	0.68802	1.37033	0.97275	0.05	0	-1.71535	3.04868
T7	T3	-0.32667	0.68802	0.67146	0.99964	0.05	0	-2.70868	2.05535
T7	T4	-0.20667	0.68802	0.4248	0.99998	0.05	0	-2.58868	2.17535
T7	T5	-1.69667	0.68802	3.48749	0.27603	0.05	0	-4.07868	0.68535
T7	T6	-1.31667	0.68802	2.7064	0.56123	0.05	0	-3.69868	1.06535
T8	T1	5.01333	0.68802	10.30488	<0.0001	0.05	1	2.63132	7.39535
T8	T2	2.99667	0.68802	6.15963	0.00895	0.05	1	0.61465	5.37868

T8	T3	2.00333	0.68802	4.11784	0.13479	0.05	0	-0.37868	4.38535
T8	T4	2.12333	0.68802	4.3645	0.09942	0.05	0	-0.25868	4.50535
T8	T5	0.63333	0.68802	1.30181	0.97935	0.05	0	-1.74868	3.01535
T8	T6	1.01333	0.68802	2.0829	0.81051	0.05	0	-1.36868	3.39535
T8	T7	2.33	0.68802	4.7893	0.05757	0.05	0	-0.05201	4.71201

[S3 Table. ANOVA for plant height]

		MeanDiff	SEM	q Value	Prob	Alpha	Sig	LCL	UCL
T2	T1	6.90667	2.44024	4.00268	0.15475	0.05	0	-1.54179	15.35513
T3	T1	6.65667	2.44024	3.8578	0.18336	0.05	0	-1.79179	15.10513
T3	T2	-0.25	2.44024	0.14488	1	0.05	0	-8.69846	8.19846
T4	T1	7.51333	2.44024	4.35427	0.1007	0.05	0	-0.93513	15.96179
T4	T2	0.60667	2.44024	0.35159	0.99999	0.05	0	-7.84179	9.05513
T4	T3	0.85667	2.44024	0.49647	0.99995	0.05	0	-7.59179	9.30513
T5	T1	10.65667	2.44024	6.17596	0.00875	0.05	1	2.20821	19.10513
T5	T2	3.75	2.44024	2.17327	0.77808	0.05	0	-4.69846	12.19846
T5	T3	4	2.44024	2.31816	0.7223	0.05	0	-4.44846	12.44846
T5	T4	3.14333	2.44024	1.82168	0.89059	0.05	0	-5.30513	11.59179
T6	T1	8.47667	2.44024	4.91256	0.04893	0.05	1	0.02821	16.92513
T6	T2	1.57	2.44024	0.90988	0.99748	0.05	0	-6.87846	10.01846
T6	T3	1.82	2.44024	1.05476	0.99381	0.05	0	-6.62846	10.26846
T6	T4	0.96333	2.44024	0.55829	0.99989	0.05	0	-7.48513	9.41179
T6	T5	-2.18	2.44024	1.2634	0.98249	0.05	0	-10.6285	6.26846
T7	T1	6.49333	2.44024	3.76314	0.20429	0.05	0	-1.95513	14.94179
T7	T2	-0.41333	2.44024	0.23954	1	0.05	0	-	8.03513

								8.86179	
T7	T3	-0.16333	2.44024	0.09466	1	0.05	0	-8.61179	8.28513
T7	T4	-1.02	2.44024	0.59113	0.99984	0.05	0	-9.46846	7.42846
T7	T5	-4.16333	2.44024	2.41281	0.68398	0.05	0	-12.6118	4.28513
T7	T6	-1.98333	2.44024	1.14942	0.98975	0.05	0	-10.4318	6.46513
T8	T1	15.03667	2.44024	8.71434	2.87E-04	0.05	1	6.58821	23.48513
T8	T2	8.13	2.44024	4.71165	0.06373	0.05	0	-0.31846	16.57846
T8	T3	8.38	2.44024	4.85654	0.05269	0.05	0	-0.06846	16.82846
T8	T4	7.52333	2.44024	4.36007	0.09997	0.05	0	-0.92513	15.97179
T8	T5	4.38	2.44024	2.53838	0.63178	0.05	0	-4.06846	12.82846
T8	T6	6.56	2.44024	3.80178	0.19553	0.05	0	-1.88846	15.00846
T8	T7	8.54333	2.44024	4.9512	0.04648	0.05	1	0.09487	16.99179

[S4 Table. ANOVA for Stem diameter]

		MeanDiff	SEM	q Value	Prob	Alpha	Sig	LCL	UCL
T2	T1	0.00667	0.16076	0.05865	1	0.05	0	-0.54992	0.56325
T3	T1	0.23333	0.16076	2.05262	0.82088	0.05	0	-0.32325	0.78992
T3	T2	0.22667	0.16076	1.99397	0.84022	0.05	0	-0.32992	0.78325
T4	T1	0.16667	0.16076	1.46616	0.96117	0.05	0	-0.38992	0.72325
T4	T2	0.16	0.16076	1.40751	0.9686	0.05	0	-0.39658	0.71658
T4	T3	-0.06667	0.16076	0.58646	0.99985	0.05	0	-0.62325	0.48992
T5	T1	0.53	0.16076	4.66238	0.06794	0.05	0	-0.02658	1.08658
T5	T2	0.52333	0.16076	4.60373	0.0733	0.05	0	-0.03325	1.07992
T5	T3	0.29667	0.16076	2.60976	0.60179	0.05	0	-0.25992	0.85325
T5	T4	0.36333	0.16076	3.19622	0.36968	0.05	0	-0.19325	0.91992
T6	T1	0.50667	0.16076	4.45711	0.08844	0.05	0	-	1.06325

								0.04992	
T6	T2	0.5	0.16076	4.39847	0.09526	0.05	0	-0.05658	1.05658
T6	T3	0.27333	0.16076	2.4045	0.68739	0.05	0	-0.28325	0.82992
T6	T4	0.34	0.16076	2.99096	0.44579	0.05	0	-0.21658	0.89658
T6	T5	-0.02333	0.16076	0.20526	1	0.05	0	-0.57992	0.53325
T7	T1	0.4	0.16076	3.51877	0.26706	0.05	0	-0.15658	0.95658
T7	T2	0.39333	0.16076	3.46013	0.28405	0.05	0	-0.16325	0.94992
T7	T3	0.16667	0.16076	1.46616	0.96117	0.05	0	-0.38992	0.72325
T7	T4	0.23333	0.16076	2.05262	0.82088	0.05	0	-0.32325	0.78992
T7	T5	-0.13	0.16076	1.1436	0.99005	0.05	0	-0.68658	0.42658
T7	T6	-0.10667	0.16076	0.93834	0.99695	0.05	0	-0.66325	0.44992
T8	T1	0.7	0.16076	6.15785	0.00897	0.05	1	0.14342	1.25658
T8	T2	0.69333	0.16076	6.09921	0.00973	0.05	1	0.13675	1.24992
T8	T3	0.46667	0.16076	4.10524	0.13686	0.05	0	-0.08992	1.02325
T8	T4	0.53333	0.16076	4.6917	0.0654	0.05	0	-0.02325	1.08992
T8	T5	0.17	0.16076	1.49548	0.95704	0.05	0	-0.38658	0.72658
T8	T6	0.19333	0.16076	1.70074	0.91973	0.05	0	-0.36325	0.74992
T8	T7	0.3	0.16076	2.63908	0.58947	0.05	0	-0.25658	0.85658

[S5 Table. ANOVA for fruit yield]

		MeanDiff	SEM	t Value	Prob	Alpha	Sig	LCL	UCL
T2	T1	1.92	0.14556	13.19007	<0.0001	0.05	1	1.37731	2.46269
T3	T1	1.52	0.14556	10.44214	<0.0001	0.05	1	0.97731	2.06269
T3	T2	-0.4	0.14556	-2.74793	0.33178	0.05	0	-0.94269	0.14269
T4	T1	1.92	0.14556	13.19007	<0.0001	0.05	1	1.37731	2.46269
T4	T2	0	0.14556	0	1	0.05	0	-0.54269	0.54269
T4	T3	0.4	0.14556	2.74793	0.33178	0.05	0	-0.14269	0.94269
T5	T1	1.95333	0.14556	13.41907	<0.0001	0.05	1	1.41065	2.49602

T5	T2	0.03333	0.14556	0.22899	1	0.05	0	-0.50935	0.57602
T5	T3	0.43333	0.14556	2.97693	0.22139	0.05	0	-0.10935	0.97602
T5	T4	0.03333	0.14556	0.22899	1	0.05	0	-0.50935	0.57602
T6	T1	2.15333	0.14556	14.79303	<0.0001	0.05	1	1.61065	2.69602
T6	T2	0.23333	0.14556	1.60296	0.97874	0.05	0	-0.30935	0.77602
T6	T3	0.63333	0.14556	4.35089	0.01378	0.05	1	0.09065	1.17602
T6	T4	0.23333	0.14556	1.60296	0.97874	0.05	0	-0.30935	0.77602
T6	T5	0.2	0.14556	1.37397	0.99711	0.05	0	-0.34269	0.74269
T7	T1	0.92	0.14556	6.32024	2.85E-04	0.05	1	0.37731	1.46269
T7	T2	-1	0.14556	-6.86983	1.06E-04	0.05	1	-1.54269	-0.45731
T7	T3	-0.6	0.14556	-4.1219	0.02213	0.05	1	-1.14269	-0.05731
T7	T4	-1	0.14556	-6.86983	1.06E-04	0.05	1	-1.54269	-0.45731
T7	T5	-1.03333	0.14556	-7.09882	<0.0001	0.05	1	-1.57602	-0.49065
T7	T6	-1.23333	0.14556	-8.47279	<0.0001	0.05	1	-1.77602	-0.69065
T8	T1	2.02	0.14556	13.87705	<0.0001	0.05	1	1.47731	2.56269
T8	T2	0.1	0.14556	0.68698	1	0.05	0	-0.44269	0.64269
T8	T3	0.5	0.14556	3.43491	0.09096	0.05	0	-0.04269	1.04269
T8	T4	0.1	0.14556	0.68698	1	0.05	0	-0.44269	0.64269
T8	T5	0.06667	0.14556	0.45799	1	0.05	0	-0.47602	0.60935
T8	T6	-0.13333	0.14556	-0.91598	1	0.05	0	-0.67602	0.40935
T8	T7	1.1	0.14556	7.55681	<0.0001	0.05	1	0.55731	1.64269

[S6 Table. ANOVA for Dry BMY]

		MeanDiff	SEM	t Value	Prob	Alpha	Sig	LCL	UCL
T2	T1	1.54	0.08529	18.05528	<0.0001	0.05	1	1.22201	1.85799
T3	T1	0.08333	0.08529	0.97702	0.99999	0.05	0	-0.23466	0.40132
T3	T2	-1.45667	0.08529	-17.0783	<0.0001	0.05	1	-1.77466	-1.13868

T4	T1	0.11667	0.08529	1.36782	0.99729	0.05	0	-0.20132	0.43466
T4	T2	-1.42333	0.08529	-16.6875	<0.0001	0.05	1	-1.74132	-1.10534
T4	T3	0.03333	0.08529	0.39081	1	0.05	0	-0.28466	0.35132
T5	T1	0.16667	0.08529	1.95403	0.86249	0.05	0	-0.15132	0.48466
T5	T2	-1.37333	0.08529	-16.1013	<0.0001	0.05	1	-1.69132	-1.05534
T5	T3	0.08333	0.08529	0.97702	0.99999	0.05	0	-0.23466	0.40132
T5	T4	0.05	0.08529	0.58621	1	0.05	0	-0.26799	0.36799
T6	T1	0.26667	0.08529	3.12646	0.1671	0.05	0	-0.05132	0.58466
T6	T2	-1.27333	0.08529	-14.9288	<0.0001	0.05	1	-1.59132	-0.95534
T6	T3	0.18333	0.08529	2.14944	0.74207	0.05	0	-0.13466	0.50132
T6	T4	0.15	0.08529	1.75863	0.94387	0.05	0	-0.16799	0.46799
T6	T5	0.1	0.08529	1.17242	0.99977	0.05	0	-0.21799	0.41799
T7	T1	0.11667	0.08529	1.36782	0.99729	0.05	0	-0.20132	0.43466
T7	T2	-1.42333	0.08529	-16.6875	<0.0001	0.05	1	-1.74132	-1.10534
T7	T3	0.03333	0.08529	0.39081	1	0.05	0	-0.28466	0.35132
T7	T4	0	0.08529	0	1	0.05	0	-0.31799	0.31799
T7	T5	-0.05	0.08529	-0.58621	1	0.05	0	-0.36799	0.26799
T7	T6	-0.15	0.08529	-1.75863	0.94387	0.05	0	-0.46799	0.16799
T8	T1	0.16667	0.08529	1.95403	0.86249	0.05	0	-0.15132	0.48466
T8	T2	-1.37333	0.08529	-16.1013	<0.0001	0.05	1	-1.69132	-1.05534
T8	T3	0.08333	0.08529	0.97702	0.99999	0.05	0	-0.23466	0.40132
T8	T4	0.05	0.08529	0.58621	1	0.05	0	-0.26799	0.36799
T8	T5	0	0.08529	0	1	0.05	0	-0.31799	0.31799
T8	T6	-0.1	0.08529	-1.17242	0.99977	0.05	0	-0.41799	0.21799

T8	T7	0.05	0.08529	0.58621	1	0.05	0	-0.26799	0.36799
