# Supplementary material for: Synergetic effect of bioslurry and charcoal co – composted fertilizers on soil properties and tomato (Solanum lycopersicum) productivity
Source: PLoS One. 2026 Jul 28;21(7):e0353951. doi: 10.1371/journal.pone.0353951 (PMC13411927; doi:10.1371/journal.pone.0353951)
Supplement: Attachment — Submitted filename: Comments 20-04-2026.docx [file pone.0353951.s002.docx]

**Manuscript ID: PONE-D-26-10820**

Title: Impacts of anaerobic cow dung bioslurry-charcoal co-composted fertilizers on tomato (Solanum lycopersicum) productivity and soil properties. The manuscript addresses an important topic but requires substantial corrections in data consistency, statistical analysis, experimental clarity, and interpretation before it can be considered for publication.

**General assessment**

This manuscript investigates the agronomic and environmental potential of co-composted anaerobic cow dung bioslurry and charcoal waste (ACDBCFs) as a fertilizer for tomato production under greenhouse conditions. The topic is relevant to sustainable agriculture, waste valorization, and soil fertility management, particularly in low-input farming systems.

The study presents experimental data including physicochemical characterization, plant growth responses, and multivariate analyses (PCA and heat map). However, despite its relevance, the manuscript currently contains several major methodological, analytical, and reporting issues that limit the reliability and reproducibility of the findings.

**Major Comments**

**1. Critical inconsistencies in units and reported values:** There are significant inconsistencies between the Abstract, Methods, and Results. For example, the Abstract reports dry biomass yield up to 0.60 kg pot⁻¹, while Table 4 shows a maximum of 0.10 kg. Similarly, yield units are inconsistently described as kg pot⁻¹ and kg m⁻². These discrepancies must be corrected throughout the manuscript. The authors should standardize units and clearly define whether results are expressed per pot, per plant, or per unit area.

**2. Unclear treatment structure and experimental description:** The treatment definitions (T1–T8) are not consistently explained across the manuscript. While Table 2 lists application rates, it does not clearly identify which treatments correspond to ACDBCF formulations, ACDB alone, or NPS fertilizer.

**3. Figure 2** partially clarifies this, but treatment identities should be explicitly stated in the tables and text to avoid ambiguity.

**4. Insufficient statistical analysis reporting:** The manuscript mentions ANOVA but does not provide: The statistical model used, blocking structure details and post hoc test used for mean separation. Additionally, extremely high coefficients of variation (e.g., CV up to 445%) suggest possible data instability or analytical errors. The statistical analysis needs to be fully revised and clearly reported.

**5. Overinterpretation of greenhouse pot experiment results:** The study is based on a short-term greenhouse pot experiment, yet the conclusions extend to broader agricultural applications and soil fertility improvements. The authors should moderate these claims and clearly acknowledge the limitations of pot experiments, including lack of field validation, limited representation of real soil conditions and absence of long-term nutrient dynamics.

**6.** Professional editing is strongly recommended.

- Table 3: Contains formatting and typographical issues (e.g., inconsistent decimals). Units should be carefully checked.
- Figures 3 & 4: Lack clarity on replication and error bars.

**7.** The manuscript would benefit from the inclusion of recent and relevant studies to strengthen the scientific context, particularly in relation to integrated soil amendments and sustainable crop productivity. For instance, Zahra et al. (2026) provide valuable insights into the role of biochar in enhancing nutrient dynamics and plant performance. Similarly, Shah et al. (2025) demonstrate the synergistic effects of combining organic compost with inorganic fertilizers on biomass production and nutrient accumulation. In addition, Jabran et al. (2024) highlight the importance of bio-based inputs in regulating crop growth and improving soil–plant interactions, which could further strengthen the discussion on sustainable nutrient management.

Incorporating these references will enhance the manuscript’s scientific depth, improve interpretation of results, and align it with current advances in sustainable agriculture research.

1. Zahra, G. E., Bareen, F. E., Pasha, M. F. K., Zahra, S., Oranab, S., Zahra, S., & Ahmad, H. M. (2026). Synergistic enhancement of heavy metal phytoextraction in okra (Abelmoschus esculentus) using biochar and chelating agents. Integrative Plant Biotechnology, 4(1), 1–11. <https://doi.org/10.55627/pbiotech.004.01.1374>
2. Shah, Z.-u.-h., Suhag, K. R., Jamro, G. M., Talpur, N. A., Rashdi, H. S., Jamali, I. A., Memon, F. N., & Khanzada, I.-u.-H. (2025). Integrated application of biocompost pressmud and chemical fertilizers to enhance biomass production and nutrient accumulation of maize. Integrative Plant Biotechnology, 3(3), 269–280. https://doi.org/10.58257/pbiotech.v3i3.1400
3. Jabran M., Ali MA, Acet T, Zahoor A, Abbas A, Arshad U, Mubashar M, Naveed M, Ghafoor A, Gao L. 2024. Growth regulation in bread wheat via novel bioinoculant formulation. BMC Plant Biology 24, 1039. https://doi.org/10.1186/s12870-024-05698-x.

**Minor Comments**

4. The title could be revise for clarity.
5. The term “acid soil” is somewhat misleading given initial soil pH (~6.43).
6. Ensure consistent use of terminology (bioslurry vs bio-slurry).
7. Clarify plant density per pot and thinning practices.
8. Explain how field application rates (t ha⁻¹) were converted to pot-level doses.
9. Improve reference formatting and relevance.
