# Supplementary material for: Synergetic effect of bioslurry and charcoal co – composted fertilizers on soil properties and tomato (Solanum lycopersicum) productivity
Source: PLoS One. 2026 Jul 28;21(7):e0353951. doi: 10.1371/journal.pone.0353951 (PMC13411927; doi:10.1371/journal.pone.0353951)
Supplement: Peer review history

# Peer review: Decision Letter 0

**DOI:** 10.1371/journal.pone.0353951.r001

**Authors:** Muhammad Amjad Ali

20 Apr 2026

PONE-D-26-10820

Impacts of anaerobic cow dung bioslurry-charcoal co-composted fertilizers on tomato (Solanum lycopersicum) productivity and soil properties

PLOS One

Dear Dr. Alayu,

Thank you for submitting your manuscript to PLOS ONE. After careful consideration, we feel that it has merit but does not fully meet PLOS ONE’s publication criteria as it currently stands. Therefore, we invite you to submit a revised version of the manuscript that addresses the points raised during the review process.

Please submit your revised manuscript by Jun 04 2026 11:59PM. If you will need more time than this to complete your revisions, please reply to this message or contact the journal office at plosone@plos.org. When you're ready to submit your revision, log on to https://www.editorialmanager.com/pone/ and select the 'Submissions Needing Revision' folder to locate your manuscript file.

Please include the following items when submitting your revised manuscript:

- A letter that responds to each point raised by the academic editor and reviewer(s). You should upload this letter as a separate file labeled 'Response to Reviewers'.
- A marked-up copy of your manuscript that highlights changes made to the original version. You should upload this as a separate file labeled 'Revised Manuscript with Track Changes'.
- An unmarked version of your revised paper without tracked changes. You should upload this as a separate file labeled 'Manuscript'.

If you would like to make changes to your financial disclosure, please include your updated statement in your cover letter. Guidelines for resubmitting your figure files are available below the reviewer comments at the end of this letter.

If applicable, we recommend that you deposit your laboratory protocols in protocols.io to enhance the reproducibility of your results. Protocols.io assigns your protocol its own identifier (DOI) so that it can be cited independently in the future. For instructions see: https://journals.plos.org/plosone/s/submission-guidelines#loc-laboratory-protocols. Additionally, PLOS ONE offers an option for publishing peer-reviewed Lab Protocol articles, which describe protocols hosted on protocols.io. Read more information on sharing protocols at https://plos.org/protocols?utm_medium=editorial-email&utm_source=authorletters&utm_campaign=protocols.

As the corresponding author, your ORCID iD is verified in the submission system and will appear in the published article. PLOS supports the use of ORCID, and we encourage all coauthors to register for an ORCID iD and use it as well. Please encourage your coauthors to verify their ORCID iD within the submission system before final acceptance, as unverified ORCID iDs will not appear in the published article. *Only*  the individual author can complete the verification step; PLOS staff *cannot*  verify ORCID iDs on behalf of authors.

We look forward to receiving your revised manuscript.

Kind regards,

Muhammad Amjad Ali, PhD

Academic Editor

PLOS One

Journal Requirements:

When submitting your revision, we need you to address these additional requirements.

1. Please ensure that your manuscript meets PLOS ONE's style requirements, including those for file naming. The PLOS ONE style templates can be found at

https://journals.plos.org/plosone/s/file?id=wjVg/PLOSOne_formatting_sample_main_body.pdf and

https://journals.plos.org/plosone/s/file?id=ba62/PLOSOne_formatting_sample_title_authors_affiliations.pdf

2. In your Methods section, please provide additional information regarding the permits you obtained for the work. Please ensure you have included the full name of the authority that approved the field site access and, if no permits were required, a brief statement explaining why.

3. Thank you for stating the following in your Competing Interests section:

“No conflict of interest”

Please complete your Competing Interests on the online submission form to state any Competing Interests. If you have no competing interests, please state "The authors have declared that no competing interests exist.", as detailed online in our guide for authors at http://journals.plos.org/plosone/s/submit-now

This information should be included in your cover letter; we will change the online submission form on your behalf.

4. When completing the data availability statement of the submission form, you indicated that you will make your data available on acceptance. We strongly recommend all authors decide on a data sharing plan before acceptance, as the process can be lengthy and hold up publication timelines. Please note that, though access restrictions are acceptable now, your entire data will need to be made freely accessible if your manuscript is accepted for publication. This policy applies to all data except where public deposition would breach compliance with the protocol approved by your research ethics board. If you are unable to adhere to our open data policy, please kindly revise your statement to explain your reasoning and we will seek the editor's input on an exemption. Please be assured that, once you have provided your new statement, the assessment of your exemption will not hold up the peer review process.

5. Please include a separate caption for each figure in your manuscript.

6. We note that Figure 1 in your submission contain [map/satellite] images which may be copyrighted. All PLOS content is published under the Creative Commons Attribution License (CC BY 4.0), which means that the manuscript, images, and Supporting Information files will be freely available online, and any third party is permitted to access, download, copy, distribute, and use these materials in any way, even commercially, with proper attribution. For these reasons, we cannot publish previously copyrighted maps or satellite images created using proprietary data, such as Google software (Google Maps, Street View, and Earth). For more information, see our copyright guidelines: http://journals.plos.org/plosone/s/licenses-and-copyright.

We require you to either (1) present written permission from the copyright holder to publish these figures specifically under the CC BY 4.0 license, or (2) remove the figures from your submission:

1. You may seek permission from the original copyright holder of Figure 1 to publish the content specifically under the CC BY 4.0 license.

We recommend that you contact the original copyright holder with the Content Permission Form (http://journals.plos.org/plosone/s/file?id=7c09/content-permission-form.pdf) and the following text:

“I request permission for the open-access journal PLOS ONE to publish XXX under the Creative Commons Attribution License (CCAL) CC BY 4.0 (http://creativecommons.org/licenses/by/4.0/). Please be aware that this license allows unrestricted use and distribution, even commercially, by third parties. Please reply and provide explicit written permission to publish XXX under a CC BY license and complete the attached form.”

Please upload the completed Content Permission Form or other proof of granted permissions as an ""Other"" file with your submission.

In the figure caption of the copyrighted figure, please include the following text: “Reprinted from [ref] under a CC BY license, with permission from [name of publisher], original copyright [original copyright year].”

2. If you are unable to obtain permission from the original copyright holder to publish these figures under the CC BY 4.0 license or if the copyright holder’s requirements are incompatible with the CC BY 4.0 license, please either i) remove the figure or ii) supply a replacement figure that complies with the CC BY 4.0 license. Please check copyright information on all replacement figures and update the figure caption with source information. If applicable, please specify in the figure caption text when a figure is similar but not identical to the original image and is therefore for illustrative purposes only.

The following resources for replacing copyrighted map figures may be helpful:

USGS National Map Viewer (public domain): http://viewer.nationalmap.gov/viewer/

The Gateway to Astronaut Photography of Earth (public domain): http://eol.jsc.nasa.gov/sseop/clickmap/

Maps at the CIA (public domain): https://www.cia.gov/library/publications/the-world-factbook/index.html and https://www.cia.gov/library/publications/cia-maps-publications/index.html

NASA Earth Observatory (public domain): http://earthobservatory.nasa.gov/

Landsat: http://landsat.visibleearth.nasa.gov/

USGS EROS (Earth Resources Observatory and Science (EROS) Center) (public domain): http://eros.usgs.gov/#

Natural Earth (public domain): http://www.naturalearthdata.com/

7. If the reviewer comments include a recommendation to cite specific previously published works, please review and evaluate these publications to determine whether they are relevant and should be cited. There is no requirement to cite these works unless the editor has indicated otherwise.

[Note: HTML markup is below. Please do not edit.]

Reviewers' comments:

Reviewer's Responses to Questions

**Comments to the Author**

1. Is the manuscript technically sound, and do the data support the conclusions?

The manuscript must describe a technically sound piece of scientific research with data that supports the conclusions. Experiments must have been conducted rigorously, with appropriate controls, replication, and sample sizes. The conclusions must be drawn appropriately based on the data presented.

Reviewer #1: Yes

Reviewer #2: Yes

**********

2. Has the statistical analysis been performed appropriately and rigorously?

Reviewer #1: Yes

Reviewer #2: Yes

**********

3. Have the authors made all data underlying the findings in their manuscript fully available?

The PLOS Data policy requires authors to make all data underlying the findings described in their manuscript fully available without restriction, with rare exception (please refer to the Data Availability Statement in the manuscript PDF file). The data should be provided as part of the manuscript or its supporting information, or deposited to a public repository. For example, in addition to summary statistics, the data points behind means, medians and variance measures should be available. If there are restrictions on publicly sharing data—e.g. participant privacy or use of data from a third party—those must be specified.

Reviewer #1: Yes

Reviewer #2: Yes

**********

4. Is the manuscript presented in an intelligible fashion and written in standard English?

PLOS ONE does not copyedit accepted manuscripts, so the language in submitted articles must be clear, correct, and unambiguous. Any typographical or grammatical errors should be corrected at revision, so please note any specific errors here.

Reviewer #1: Yes

Reviewer #2: Yes

**********

5. Review Comments to the Author

Please use the space provided to explain your answers to the questions above. You may also include additional comments for the author, including concerns about dual publication, research ethics, or publication ethics. (Please upload your review as an attachment if it exceeds 20,000 characters)

Reviewer #1: The manuscript with ID: PONE-D-26-10820, title “Impacts of anaerobic cow dung bioslurry-charcoal co-composted fertilizers on tomato (Solanum lycopersicum) productivity and soil properties”. The study addresses an important and timely topic by exploring the reuse of locally available wastes (bioslurry and charcoal) for improving soil fertility and tomato productivity. The integration of agronomic, physicochemical, and multivariate analyses is commendable, and the study has clear relevance for sustainable agriculture, particularly in resource-constrained settings such as Ethiopia. The work demonstrates promising findings and has the potential to contribute meaningfully to the field after appropriate revisions. However, the manuscript requires major revision before it can be considered for publication. The following comments are provided to help strengthen the rigor, clarity, and overall quality of the study:

Major Comments

1. The title should be revised to replace the term “charcoal” with “biochar”, as this is the more scientifically appropriate and widely accepted terminology in the literature.

2. The experimental design lacks clarity, particularly regarding the definition of treatments (T1–T8). A clear mapping between treatment codes and compost formulations should be provided to avoid confusion.

3. The basis for dosage calculations (t ha⁻¹ to kg pot⁻¹) is not sufficiently explained. It is recommended that the conversion procedure be clearly shown to ensure reproducibility.

4. The use of a randomized complete block design (RCBD) is stated, but the blocking factor is not described. This should be clarified, or the design reconsidered if blocking was not meaningfully implemented.

5. The replication level (n = 3) is relatively low for greenhouse experiments. A justification (e.g., statistical power or precedent) should be included.

6. Some measured values, particularly electrical conductivity (EC), appear unrealistic (e.g., values exceeding 1000 mS/cm). These should be carefully rechecked, and units should be standardized (preferably dS/m).

7. The statistical analysis section is insufficiently detailed. The statistical model, assumptions (normality, homogeneity), and post-hoc tests should be clearly described.

8. The reporting of p-values (mostly as p < 0.001) appears overly generalized. Exact p-values or appropriate ranges should be reported where possible.

9. The very high coefficient of variation (CV) values reported in some tables (e.g., >100%) raise concerns about data reliability and should be verified.

10. The interpretation of PCA results tends to imply causation, whereas only associations can be inferred. The language should be revised accordingly.

11. The manuscript merges results and discussion, leading to redundancy and reduced clarity. It is recommended that these sections be separated.

12. The manuscript would benefit from incorporating recent literature on the broader benefits of biochar, particularly its role not only in soil fertility improvement but also in mitigating biotic and abiotic stresses. For instance, studies such as Fayyaz et al. (2024) 10.21162/PAKJAS/24.30 and related recent work (https://doi.org/10.1007/s42773-025-00521-8; https://doi.org/10.55627/pbiotech.002.01.0972) demonstrate the effectiveness of biochar in suppressing root-knot nematodes and enhancing plant resilience. Integrating such references would strengthen the discussion and better position the study within the current scientific context.

13. The short-term greenhouse nature of the study limits the generalizability of the conclusions. Claims regarding field-scale applicability should be moderated.

14. The data availability statement should be more precise (e.g., repository, DOI), as per journal requirements.

15. Some treatments (e.g., T8 with very low application rate) appear inconsistent and require justification or correction.

16. The manuscript would benefit from clear reporting of variability (mean ± SD or SE) across all measured parameters.

Minor Language Comments

1. Several sentences are overly long and complex, and should be broken into shorter, clearer statements for readability.

2. There is inconsistent use of articles (“a”, “the”), particularly in the introduction and methods sections.

3. The phrase “in the same argument” should be revised to “similarly” or “in line with previous studies”.

4. Some subject–verb agreement errors are present (e.g., “results shows” instead of “results show”).

5. The manuscript contains redundant wording, such as repeated phrases like “promising results,” which should be minimized.

6. Tense usage is inconsistent, especially between past and present tense in the Results section; past tense should be used for reporting findings.

7. Several typographical errors and spacing issues are present (e.g., missing spaces between units and values).

8. Scientific names such as Solanum lycopersicum should be italicized consistently throughout the manuscript.

9. Units are sometimes incorrectly formatted or inconsistent (e.g., mS/cm vs dS/m); standard SI formatting should be followed.

10. Some sentences lack clarity and precision, particularly in the discussion, and should be revised for conciseness and academic tone.

Reviewer #2: This study has a strong conceptual foundation and practical relevance. However, data accuracy and clarity must be prioritized. Addressing the issues above will significantly improve the manuscript’s credibility and increase its chances of acceptance. The statistical analysis requires substantial improvement by detailing the model, assumptions, and post hoc tests, and by reassessing data variability where coefficients of variation are unusually high. Compost characterization should be strengthened by reporting key parameters such as C:N ratio and maturity indicators to support agronomic interpretations. The discussion should be revised to avoid overgeneralization from short-term greenhouse results and include a more balanced evaluation of potential risks such as high pH and salinity effects. Figures and tables, especially Table 5, should be carefully revised for accuracy, clarity, and proper labeling, including sample sizes and error bar explanations. **For further comments, see the attached file, please.**

**********

6. PLOS authors have the option to publish the peer review history of their article (what does this mean?). If published, this will include your full peer review and any attached files.

If you choose “no”, your identity will remain anonymous but your review may still be made public.

**Do you want your identity to be public for this peer review?**  For information about this choice, including consent withdrawal, please see our Privacy Policy.

Reviewer #1: **Yes:** Amjad Abbas

Reviewer #2: No

**********

[NOTE: If reviewer comments were submitted as an attachment file, they will be attached to this email and accessible via the submission site. Please log into your account, locate the manuscript record, and check for the action link "View Attachments". If this link does not appear, there are no attachment files.]

To ensure your figures meet our technical requirements, please review our figure guidelines: https://journals.plos.org/plosone/s/figures

You may also use PLOS’s free figure tool, NAAS, to help you prepare publication quality figures: https://journals.plos.org/plosone/s/figures#loc-tools-for-figure-preparation.

NAAS will assess whether your figures meet our technical requirements by comparing each figure against our figure specifications.

Attachment Submitted filename: Comments 20-04-2026.docx

# Author comment: Author response to Decision Letter 1

**DOI:** 10.1371/journal.pone.0353951.r002

11 Jun 2026

We express our sincere gratitude for your insightful and constructive comments on our manuscript. Your valuable feedback has significantly helped us strengthen the scientific quality, clarity, and precision of our work. We have comprehensively addressed all major and minor comments raised. To facilitate the review process, all modifications, data corrections, and textual updates have been explicitly highlighted in red within the revised manuscript. Detailed point-by-point responses to each of your specific concerns are provided.

# Editor report: Decision Letter 1

**DOI:** 10.1371/journal.pone.0353951.r003

**Authors:** Muhammad Amjad Ali

2 Jul 2026

Synergetic effect of bioslurry and charcoal co – composted fertilizers on soil properties and tomato (Solanum lycopersicum) productivity

PONE-D-26-10820R1

Dear Dr. Alayu,

We’re pleased to inform you that your manuscript has been judged scientifically suitable for publication and will be formally accepted for publication once it meets all outstanding technical requirements.

Within one week, you’ll receive an e-mail detailing the required amendments. When these have been addressed, you’ll receive a formal acceptance letter and your manuscript will be scheduled for publication.

An invoice will be generated when your article is formally accepted. Please note, if your institution has a publishing partnership with PLOS and your article meets the relevant criteria, all or part of your publication costs will be covered. Please make sure your user information is up-to-date by logging into Editorial Manager at Editorial Manager® and clicking the ‘Update My Information' link at the top of the page. For questions related to billing, please contact billing support.

If your institution or institutions have a press office, please notify them about your upcoming paper to help maximize its impact. If they’ll be preparing press materials, please inform our press team as soon as possible -- no later than 48 hours after receiving the formal acceptance. Your manuscript will remain under strict press embargo until 2 pm Eastern Time on the date of publication. For more information, please contact onepress@plos.org.

Kind regards,

Muhammad Amjad Ali, PhD

Academic Editor

PLOS One

Additional Editor Comments (optional):

Reviewers' comments:

# Editor report: Acceptance letter

**DOI:** 10.1371/journal.pone.0353951.r004

**Authors:** Muhammad Amjad Ali

PONE-D-26-10820R1

PLOS One

Dear Dr. Alayu,

I'm pleased to inform you that your manuscript has been deemed suitable for publication in PLOS One. Congratulations! Your manuscript is now being handed over to our production team.

At this stage, our production department will prepare your paper for publication. This includes ensuring the following:

* All references, tables, and figures are properly cited

* All relevant supporting information is included in the manuscript submission,

* There are no issues that prevent the paper from being properly typeset

You will receive further instructions from the production team, including instructions on how to review your proof when it is ready. Please keep in mind that we are working through a large volume of accepted articles, so please give us a few days to review your paper and let you know the next and final steps.

Lastly, if your institution or institutions have a press office, please let them know about your upcoming paper now to help maximize its impact. If they'll be preparing press materials, please inform our press team within the next 48 hours. Your manuscript will remain under strict press embargo until 2 pm Eastern Time on the date of publication. For more information, please contact onepress@plos.org.

You will receive an invoice from PLOS for your publication fee after your manuscript has reached the completed accept phase. If you receive an email requesting payment before acceptance or for any other service, this may be a phishing scheme. Learn how to identify phishing emails and protect your accounts at https://explore.plos.org/phishing.

If we can help with anything else, please email us at customercare@plos.org.

Thank you for submitting your work to PLOS ONE and supporting open access.

Kind regards,

PLOS ONE Editorial Office Staff

on behalf of

Dr. Muhammad Amjad Ali

Academic Editor

PLOS One
